# An Illustration of the Exploratory Structural Equation Modeling (ESEM) Framework on the Passion Scale

**DOI:** 10.3389/fpsyg.2017.01968

**Published:** 2017-11-07

**Authors:** István Tóth-Király, Beáta Bõthe, Adrien Rigó, Gábor Orosz

**Affiliations:** ^1^Doctoral School of Psychology, ELTE Eötvös Loránd University, Budapest, Hungary; ^2^Department of Personality and Health Psychology, Institute of Psychology, ELTE Eötvös Loránd University, Budapest, Hungary; ^3^Department of Clinical Psychology and Addiction, Institute of Psychology, ELTE Eötvös Loránd University, Budapest, Hungary; ^4^Department of Social Psychology, Institute of Psychology, ELTE Eötvös Loránd University, Budapest, Hungary; ^5^Institute of Cognitive Neuroscience and Psychology, Research Centre for Natural Sciences, Hungarian Academy of Sciences (MTA), Budapest, Hungary

**Keywords:** differential item functioning (DIF), dualistic model of passion (DMP), exploratory structural equation modeling (ESEM), Hungarian version, hybrid modeling approach, measurement invariance, multiple indicators multiple causes (MIMIC) model, passion scale

## Abstract

While exploratory factor analysis (EFA) provides a more realistic presentation of the data with the allowance of item cross-loadings, confirmatory factor analysis (CFA) includes many methodological advances that the former does not. To create a synergy of the two, exploratory structural equation modeling (ESEM) was proposed as an alternative solution, incorporating the advantages of EFA and CFA. The present investigation is thus an illustrative demonstration of the applicability and flexibility of ESEM. To achieve this goal, we compared CFA and ESEM models, then thoroughly tested measurement invariance and differential item functioning through multiple-indicators-multiple-causes (MIMIC) models on the Passion Scale, the only measure of the Dualistic Model of Passion (DMP) which differentiates between harmonious and obsessive forms of passion. Moreover, a hybrid model was also created to overcome the drawbacks of the two methods. Analyses of the first large community sample (*N* = 7,466; 67.7% females; *M*_*age*_ = 26.01) revealed the superiority of the ESEM model relative to CFA in terms of improved goodness-of-fit and less correlated factors, while at the same time retaining the high definition of the factors. However, this fit was only achieved with the inclusion of three correlated uniquenesses, two of which appeared in previous studies and one of which was specific to the current investigation. These findings were replicated on a second, comprehensive sample (*N* = 504; 51.8% females; *M*_*age*_ = 39.59). After combining the two samples, complete measurement invariance (factor loadings, item intercepts, item uniquenesses, factor variances-covariances, and latent means) was achieved across gender and partial invariance across age groups and their combination. Only one item intercept was non-invariant across both multigroup and MIMIC approaches, an observation that was further corroborated by the hybrid model. While obsessive passion showed a slight decline in the hybrid model, harmonious passion did not. Overall, the ESEM framework is a viable alternative of CFA that could be used and even extended to address substantially important questions and researchers should systematically compare these two approaches to identify the most suitable one.

## Introduction

Confirmatory factor analysis (CFA; Jöreskog, 1969) has been at the heart of psychometric research since its inception and quickly became a default, “go-to” method in psychometrics due to the methodological advances associated with it (e.g., goodness-of-fit, estimation of different models, inclusion of method factors or correlated uniquenesses) relative to exploratory factor analysis (EFA). Another important property—and drawback as we will demonstrate—of CFA, compared to EFA, is that items are only allowed to load on their main factors, whereas cross-loadings on the other factors are set to zero. On the other hand, EFA freely estimates all cross-loadings (Marsh et al., 2009; Morin et al., 2013). These all might contribute to the perception that EFA is less useful than or even inferior to CFA.

Although the popularity and usefulness of CFA could be seen as a motivation to create more parsimonious measurement models, these models and items more often than not include a certain level of systematic measurement error in the form of cross-loadings. Given that items are rarely pure indicators of their corresponding constructs, they are fallible in nature, thus at least some degree of construct-relevant association can be expected between items and the non-target, yet conceptually-related constructs (Morin et al., 2016). When non-zero cross-loadings are present and unexpressed at the same time, such restrictive constraints (i.e., items can only load on one factor) could inflate the associations between the factors as the misspecified cross-loadings could only be expressed through these factorial associations. Indeed, recent review of simulation studies (Asparouhov et al., 2015) showed that even small cross-loadings (as small as 0.100) should be explicitly taken into account, otherwise, parameter estimates could be inflated and thus biased. Moreover, the goodness-of-fit of the models and the discriminant validity of the factors could also be undermined by these overly restrictive specifications (Marsh et al., 2010, 2014).

**Figure 1:**
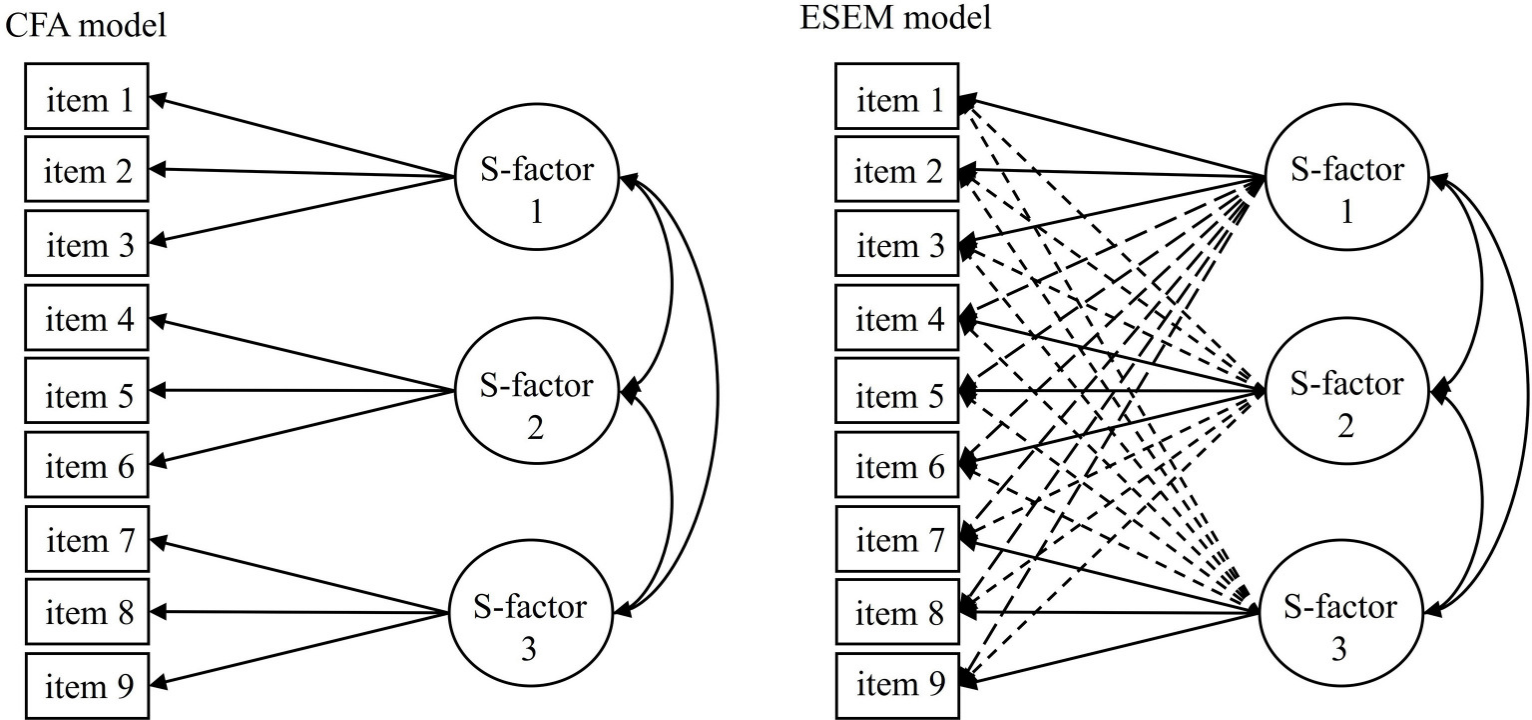
Simplified representations of the estimated models. CFA, confirmatory factor analysis; ESEM, exploratory structural equation modeling; S-factor, specific factors. Full one-headed arrows represent main factor loadings, dashed one-headed arrows represent cross-loadings, two-headed arrows represent correlations.

To overcome these serious limitations, the Exploratory Structural Equation Modeling (ESEM) framework (Asparouhov and Muthén, 2009; Marsh et al., 2014) has been developed which incorporates the advantages of the less restrictive EFA (i.e., allowing cross-loadings) and the more advanced CFA (i.e., goodness-of-fit or multigroup models) at the same time, providing a synergy that is “the best of both worlds” and can adequately account for complex measurement models (see Figure 1 for a simplistic visual representation). Generally, ESEM showed to result in improved model fit as well as deflated inter-factor correlations that, in turn, improve the discriminant validity of the factors as well as providing a more realistic representation of the data (Morin and Maïano, 2011; Morin et al., 2013; Arens and Morin, 2016; Tóth-Király et al., 2017a). Indeed, the superiority of ESEM is now well-established from a variety of studies within the field of SDT in relation to, for instance, academic (Guay et al., 2015; Tóth-Király et al., 2017c), and work (Howard et al., 2017) motivations as well as need satisfaction (Sànchez-Oliva et al., 2017). In order to demonstrate the flexibility and strength of this framework, we analyzed responses from two separate samples to the Passion Scale, the only instrument of the Dualistic Model of Passion (DMP).

### An illustrative example: on the dualistic model of passion and the passion scale

Over the last decade, research on the field of passion has boomed with the introduction of the DMP (Vallerand et al., 2003; Vallerand, 2015) stemming from the Self-Determination Theory (Deci and Ryan, 1985; Ryan and Deci, 2017). The DMP defines passion as an inclination toward an object, person, or activity that one likes (or even loves), spends a large amount of time and energy with it and finds it important. Additionally, two forms of passion can be differentiated that are qualitatively different from one another as a result of the process of internalization that takes place during activity engagement (e.g., Deci and Ryan, 1985). The first form of the DMP is *harmonious passion* (HP) which develops when autonomous and voluntary internalization occurs, thus the activity is freely engaged and incorporated into one's identity, without any inter- or intra-personal contingencies. In this case, the individual is in control of the activity. Although engaging in this activity takes up a significant amount of time, it is not overwhelming to the individual, leading to balance with other aspects of life and one's identity. Moreover, HP is predominantly associated with positive and adaptive outcomes (Vallerand, 2015). The second form of the DMP is *obsessive passion* (OP) which is rooted in a controlled internalization process where inter- or intra-personal contingencies are attached to the activity engagement, such as the maintenance of self-esteem or social acceptance (Lafrenière et al., 2011). Due to these external and/or internal contingencies, the individual loses control over the activity and feels an uncontrollable pressure to engage in it, often indirectly creating conflicts with other aspects of life. Finally, experiences of OP are often associated with negative or maladaptive outcomes (Vallerand, 2015). Despite an abundance of research focusing on the possible determinant and outcomes of passion (for a meta-analysis, see Curran et al., 2015 and for a detailed review, see Vallerand, 2015), only a couple of studies (Marsh et al., 2013; Schellenberg et al., 2014; Chamarro et al., 2015) conducted detailed examinations on the instrument measuring this construct, namely the Passion Scale.

Within the passion research, ESEM has already been demonstrated as a preferable method compared to CFA. The study of Marsh et al. (2013) was the first that evaluated the construct validity of the Passion Scale in relation to a variety of activities with the comparison of CFA and ESEM models and concluded that ESEM resulted in substantially better fit and more differentiated (i.e., less correlated) factors. These findings have been corroborated by the studies of Schellenberg et al. (2014) and of Chamarro et al. (2015) in relation to sport and exercise. Building on these studies, in the following, we illustrate the usefulness of ESEM framework as it allows the application of advanced statistical methods such as tests of measurement invariance and differential item functioning which is of major relevance to the present investigation.

### Measurement invariance and differential item functioning (DIF)

A critical point in the assessment of psychological constructs and instruments is whether they could be used among individuals with different background characteristics or at different timepoints. If the instrument (and the measurement properties) at hand behave differently in different subgroups of the population, then measurement biases could occur, leading to impossible and/or invalid comparisons. Contrarily, if findings are similar in different subgroups, then it becomes possible to generalize our findings. In practice, these assumptions could easily be inspected with tests of measurement invariance (Meredith, 1993; Vandenberg and Lance, 2000; Millsap, 2011).

Generally, based on the above-mentioned papers, there are six levels of invariance that are of key importance in these investigations. *Configural invariance* assumes that groups hold the same conceptual framework (i.e., the same factor structure) without any equality constraints on any parameters. Failure to achieve this initial level would mean that the constructs themselves differ. *Weak or metric invariance* posits the equivalence of factor loadings whose achieving is important in comparing factor correlations and relations to other constructs across groups. *Strong or scalar invariance* refers to the invariance of item intercepts and posits that members of different groups have similar item scores when the construct in question is held at the same level (i.e., group-based differences are consistent both in direction and magnitude). If this level of invariance is not achieved, then latent means cannot be compared and one can suspect the presence of DIF (i.e., response bias at the item level)*. Strict or residual invariance* tests the invariance of measurement errors across groups and is the prerequisite of manifest score comparisons. Furthermore, the equivalence of latent variances-covariances and latent means can also be examined. While the first four steps investigate the presence of measurement biases and differences, the last two steps investigate the presence of group-based differences on the level of variance, covariances, and means. The taxonomy of Marsh et al. (2009) further expanded these tests by including a total of 13 partially nested invariance models that are various combinations of the preceding ones and allow for a more thorough investigation. This taxonomy is particularly relevant for the present investigation as Marsh et al. (2013) as well as Schellenberg et al. (2014) have already demonstrated the taxonomy's usefulness in relation to passion and groups based on gender, language, and type of activity.

As for continuous variables, such as age, multiple-indicators-multiple-causes (MIMIC) models could also be pursued. MIMIC models are basically regression models where latent factors can be regressed on a diverse range of predictors. In relation to passion, Marsh et al. (2013) demonstrated through MIMIC that OP declines with age (a linear effect), but starts to flatten-out and then level off after a certain age (a quadratic effect), whereas HP was not affected by age. These are in line with the findings of Chamarro et al. (2015) to some extent as they have identified linear declines in for both HP and OP (without quadratic effect). However, a limitation of these findings is that DIF in relation to age was not tested; hence, it is possible that the predictor (i.e., age) has a unique effect on the items that cannot be fully explained by its effect on the latent variable. Researchers may have two options with continuous variables such as age: the first is to leave it as continuous and use MIMIC models to test its effect; the second is to transform age into discrete categories and test measurement invariance. As we will demonstrate, both methods have their own flaws; however, these could be amended by integrating the two methods into a single hybrid model (Marsh et al., 2006, 2013).

### The present investigation

Our main objective was to illustrate the flexibility and usefulness of the ESEM framework in relation to the Passion Scale. To this end, we first examined the factor structure of the Passion Scale with CFA and ESEM on a large community sample, then compared to two solutions to choose the most appropriate one. Based on previous studies (e.g., Marsh et al., 2013; Schellenberg et al., 2014), we expected the ESEM solution to fit the data better. The same procedure was performed on an independent comprehensive sample to assess the extent to which our findings can be replicated. After combining the two samples, we then extended the ESEM model to test measurement invariance across several group configurations (gender, age, and gender × age), evaluated the potential linear and quadratic effects of age through MIMIC models, and then combined the two methods by adding the MIMIC age effects to the gender × age invariance model.

## Materials and methods

### Participants and procedure

#### Sample 1

The first study relied on data from a total of 7,466 Hungarian adults (5047 female, 67.7%) who were aged between 18 and 74 (*M* = 26.01; *SD* = 8.43). For Sample 1, several samples with previously published and unpublished data were combined which has never been used for the psychometric investigation of the Passion Scale. Participants filled out the Passion Scale in relation to the following activities: Facebook use, series watching, learning new things, dance, playing Pokémon Go, smartphone gaming, online gaming, and sex (see Appendix 1 in the Supplementary Materials for more details). Participants were recruited through various websites, mailing lists, and online forums and filled out the questionnaires online. Before starting the questionnaire, they were first informed about the aim and the topic of the study. If they were inclined to participate, they had to approve an informed consent by checking a box; otherwise, they were excluded and their responses were recorded as finished. Therefore, the study was carried out with the adequate understanding and consent of the participants and was approved by the University Research Ethics Committee, while following the guidelines of the Declaration of Helsinki.

#### Sample 2

The second study relied on a comprehensive sample of 504 Hungarian adults who use Internet at least once a week. This sample was recruited with the help of a research market company in May 2017 using a multiple-step, proportionally stratified, probabilistic sampling method (see Tóth-Király et al., 2017a for more details on the sampling procedure) and was proportionally representative in terms of gender (51.8% female), age (18–60 years; *M* = 39.59 years; *SD* = 12.03 years), education (19.8%: primary; 58.3%: secondary; 21.8%: higher) and place of residence (20.2%: capital city; 19.6%: county capitals; 31.9%: cities; 28.2%: country). Participants reported their employment status as full-time (59.7%), part-time (8.9%), occasional (5.6%), and unemployed (25.8%). They were asked to mention an activity that they love, that spend time and energy with and that is important and valuable for them and then completed the Passion Scale with respect to that particular activity. Procedure was the same as in Study 1.

## Materials

### Passion scale

This measure (Vallerand et al., 2003; Marsh et al., 2013; Vallerand, 2015) assesses the level of passion one has for a certain activity on the basis of two dimensions: harmonious passion (six items, e.g., “My activity is in harmony with other things that are part of me.”) and obsessive passion (six items, e.g., “I have the impression that my activity controls me.”). Respondents indicated their level of agreement on a seven-point scale (1 = not agree at all; 7 = very strongly agree). A standardized back-translation procedure (Hambleton and Kanjee, 1995; Beaton et al., 2000) was followed to obtain the final Hungarian version (see Appendix 2 in the Supplementary Materials).

### Statistical analyses

#### Preliminary analyses

As data gathering was performed in an online setting, no missing responses were present. Prior to the analyses, data was investigated on the total sample for univariate normality through the inspection of skewness and kurtosis values; and multivariate normality through Mardia's two-sided test of fit for skewness and kurtosis (Wang and Wang, 2012). For univariate normality, considering the guidelines of Muthén and Kaplan (1985) with a ±1 threshold, neither skewness (ranging from −1.03 to +1.61), nor kurtosis (ranging −1.12 to +2.04) values suggested that the data has univariate normality. This observation was supported by the statistically significant Mardia's test, indicating that the assumption of multivariate normality was violated.

#### Factorial structure

All analyses were performed with Mplus 7.4 (Muthén and Muthén, 1998–2015) and estimated with the robust maximum likelihood estimator (MLR) which provides standard errors and tests of model fit that are robust to the non-normality of the data. This estimator is also preferred when there are five or more answer categories (Rhemtulla et al., 2012) such as in the present case. The first phase of the analyses included the examination of the Passion Scale through the comparison of CFA and ESEM model, as recommended by Marsh et al. (2009). As per typical CFA specification, items only loaded on their respective factor, while cross-loadings were constrained to zero. In ESEM, items loaded on their main factors, whereas cross-loadings were “targeted,” but not forced, to be as close to zero as possible with the oblique target rotation procedure (Browne, 2001). Based on previous studies (Marsh et al., 2013; Schellenberg et al., 2014; Chamarro et al., 2015), we expected that the inclusion of at least two correlated uniquenesses (CU) would be necessary given the wording of the items. Nevertheless, we first tested models without CUs. Sample input files are available in Appendix 3 in the Supplementary Materials. When interpreting the magnitude of the factor loadings, the guidelines of Comrey and Lee (2013) were applied: excellent above 0.71, very good between 0.63 and 0.70, good between 0.55 and 0.62, fair between 0.44 and 0.33, and poor below 0.32.

Another particularly important issue relates to the inclusion of a priori correlated uniquenesses (CUs; i.e., covariances between the error terms of two different items). While the *ad-hoc* inclusion of CUs should generally be avoided (Marsh et al., 2010), there are certain cases when these are acceptable (Cole et al., 2007; Marsh, 2007). Examining four previous studies on the Passion Scale revealed that all included at least two CUs in their final measurement models. More specifically, Marsh et al. (2013) had CUs between HP1-HP8 and OP7-OP9; Schellenberg et al. (2014) also identified two CUs between HP1–HP10 and OP2–OP4. Similarly, Zhao et al. (2015) included two CUs between items HP1–HP10 and OP7–OP9. Finally, the study of Chamarro et al. (2015) included a total of three CUs (HP1–HP8, OP7–OP9, OP2–OP4). While we did not formulate any specific hypotheses as to which ones should be included, we expected that at least two CUs would be necessary. For this reason, we chose to observe modification indices of the CU-less models and examine whether the necessity to include any of the above-mentioned previous CUs on a step by step, iterative basis (Oort, 1998) is replicated in our study. Without blindly including any, we also examined the content of the target items.

#### Measurement invariance

In the second phase, the measurement invariance of the most optimal measurement model was tested across the samples from the two studies to verify the replicability of the final model. Invariance tests were performed based on the extended taxonomy of Marsh et al. (2009, see also Morin et al., 2013) including a total of 13 levels of invariance with different combinations of parameters being constrained to equal. However, there are six levels that of key importance in the measurement invariance literature (Meredith, 1993; Morin et al., 2016): configural invariance, weak (metric) invariance, strong (scalar) invariance, strict (residual) invariance, latent variance-covariance invariance, and latent means invariance. Were strong measurement invariance achieved, the two samples then would be combined to maximize the available sample size when testing measurement invariance as a function of gender, age, and their interaction (gender × age). As passion is not a personality variable, we opted not to create groups based on typical interpretations of young adulthood (i.e., between 15 and 30), middle age (i.e., between 31 and 60) and older age (i.e., between 61 and 99), but instead trisected the full sample into three groups. This process resulted in three groups and age categories: 18–21 (*n* = 2,477), 22–25 (*n* = 2,563), and 26–74 (*n* = 2,930).

#### Differential item functioning (DIF)

Tests of invariance and DIF are rather complex with continuous variables such as age compared to variables with distinct categories (such as gender). One of the possible approaches is to create categorical variables from the continuous ones (as above). Although it allows for a more thorough and rigorous invariance testing, it has problems inherent to the suboptimal transformation of continuous variables which could potentially result in information loss. In similar situations, (MIMIC) models can be pursued (Morin et al., 2013, 2016). Therefore, in the third phase, building on the most invariant gender model, DIF was tested as a function of age within both gender groups where the factors were regressed on the linear and quadratic components of age (i.e., age and age^2^) as well. After standardizing age, three MIMIC models were compared (Morin et al., 2013): (1) a null effect model where the predictors (age and age^2^) have no effect on neither the items, nor the factors; (2) a saturated model where paths from the predictors to the items are freely estimated, but paths to the factors are fixed to zero; and (3) a factors-only model where paths to the factors are freely estimated, but paths to the items are constrained to zero. The comparison of the null and saturated models tests the effect of the predictors on the individual items, while the comparison of the saturated and factors-only model reveals whether these effects can be fully explained by the effects on the latent factors (i.e., the presence or absence of DIF).

#### Hybrid approach of multiple-group and mimic models

Although the multigroup solution has a disadvantage due to variable transformation, the MIMIC model is not without limitations either. More specifically, only the invariance of factor means and item intercepts can be examined (but still assumes the invariance of factor loadings and uniquenesses which cannot directly be tested). To solve this shortcoming, Marsh et al. (2006) introduced a hybrid model in which both approaches are integrated for greater precision by adding the MIMIC age effects (i.e., age and age^2^) to the multigroup model (i.e., gender × age). Moreover, this hybrid approach has already been used in conjunction with ESEM to evaluate the potential information loss as a result of transforming continuous variables (Marsh et al., 2013), making it particularly useful for the present investigation.

#### Model assessment

In interpreting the results, we relied on a combination of common goodness-of-fit indices due to the fact that they provide different information about the measurement models (Brown, 2015): the comparative fit index (CFI), the Tucker-Lewis Index (TLI), and the root mean square error of approximation (RMSEA). We considered both adequate and excellent thresholds for these fit indices (Hu and Bentler, 1999; Marsh et al., 2004, 2005; Marsh, 2007) as strictly adhering to the more conservative “golden rules” could lead to erroneous results (Chen et al., 2008; Heene et al., 2011; Perry et al., 2015). Thus, as rough guidelines, CFI and TLI values >0.90 and 0.95 and considered adequate and excellent, respectively, while RMSEA values smaller than 0.08 and 0.06 indicate acceptable and excellent model fit. Although we report the robust chi-square (χ^2^) test of exact fit as well, it has to be noted that it tends to be oversensitive to sample size and minor model misspecifications. As for model comparison, changes (Δ) in these goodness-of-fit indices were observed with lack of invariance being present if CFI and TLI decreases are at least 0.010 or higher or RMSEA increases are at least 0.015 or higher (Cheung and Rensvold, 2002; Chen, 2007). It is also worth noting that TLI and RMSEA are corrected for parsimony (i.e., more parsimonious models can fit the data better than less parsimonious ones) as opposed to CFI, which is monotonic to complexity (i.e., more complex models always fit better than less complex ones). This is of major importance given that typically more parameters as estimated in ESEM than in CFA (Marsh et al., 2009; Morin et al., 2013). Therefore, based on previous suggestions (Marsh, 2007; Marsh et al., 2013), we put a larger emphasis on TLI and RMSEA in model comparisons. However, we want to reinforce that these should only be seen as rough guidelines that one should take into account as well as the statistical and theoretical conformity of the findings (Marsh et al., 2004, 2005; Morin et al., 2016).

## Results

### Sample 1: measurement structure of the passion scale—ESEM vs. CFA

Goodness-of-fit indices for this study are presented in the top section of Table 1, while standardized parameter estimates are available on the left side of Table 2. Although we expected the necessary inclusion of correlated uniquenesses between a subset of items, we examined the two-factor CFA and ESEM models without these modifications as a starting point to see whether the same pair of items requires CUs as in previous studies. Both CU-less CFA and ESEM solutions had unsatisfactory model fit as apparent by the fit indices. The inspection of modification indices for both solutions suggested that the inclusion of three correlated uniquenesses (OP7–OP9, HP1–HP10, and OP4–OP12) would improve model fit substantially which were included on a step by step basis, starting with the pair with the highest modification indices. These modifications resulted in still unsatisfactory fit for the CFA solution, and adequate fit for the ESEM one^1^. However, the appropriate model should not only be chosen based on fit indices, but it should be complemented by the examination of parameter estimates and theoretical conformity as well (Morin et al., 2016).

**Table 1:** Goodness-of-fit statistics for the estimated models on the Passion Scale.

**Model**	**χ^2^**	**df**	**CFI**	**TLI**	**RMSEA**	**RMSEA 90% CI**
**SAMPLE 1**						
1. CFA (no CU)	5494.047	53	0.846	0.808	0.117	0.115–0.120
2. ESEM (no CU)	3447.126	43	0.904	0.852	0.103	0.100–0.106
3. CFA (1 CU)^a^	4749.137	52	0.867	0.831	0.110	0.107–0.113
4. ESEM (1 CU)^a^	2626.636	42	0.927	0.885	0.091	0.088–0.094
5. CFA (2 CUs)^b^	3895.072	51	0.891	0.859	0.100	0.098–0.103
6. ESEM (2 CUs)^b^	2025.023	41	0.944	0.910	0.081	0.078–0.084
7. CFA (3 CUs)^c^	3686.478	50	0.897	0.864	0.099	0.096–0.101
8. ESEM (3 CUs)^c^	1775.742	40	0.951	0.919	0.076	0.073–0.079
**SAMPLE 2**						
1. CFA (no CU)	350.419	53	0.831	0.790	0.106	0.095–0.116
2. ESEM (no CU)	196.050	43	0.913	0.867	0.084	0.072–0.096
3. CFA (1 CU)^a^	287.359	52	0.866	0.830	0.095	0.084–0.106
4. ESEM (1 CU)^a^	127.600	42	0.951	0.924	0.064	0.051–0.076
5. CFA (2 CUs)^b^	219.322	51	0.904	0.876	0.081	0.070–0.092
6. ESEM (2 CUs)^b^	86.804	41	0.974	0.958	0.047	0.033–0.061
7. CFA (3 CUs)^c^	196.833	50	0.917	0.890	0.076	0.065–0.088
8. ESEM (3 CUs)^c^	68.561	40	0.984	0.973	0.038	0.022–0.052

CFA, confirmatory factor analysis; ESEM, exploratory structural equation modeling; χ^2^, Robust chi-square test of exact fit; df, Degrees of freedom; CFI, Comparative fit index; TLI, Tucker-Lewis index; RMSEA, Root mean square error of approximation; 90% CI, 90% confidence interval of the RMSEA; CU, correlated uniqueness;

a correlated uniqueness between OP7 and OP9;

b correlated uniqueness between HP1 and HP10;

c *correlated uniqueness between OP4 and OP12*.

**Table 2:** Standardized parameter estimates for the CFA and ESEM solutions of the Passion Scale in study 1 and study 2.

**Items**	**Study 1 (*****N*** = **7466)**						**Study 2 (*****N*** = **504)**					
	**CFA**			**ESEM**			**CFA**			**ESEM**		
	**HP (λ)**	**OP (λ)**	**δ**	**HP (λ)**	**OP (λ)**	**δ**	**HP (λ)**	**OP (λ)**	**δ**	**HP (λ)**	**OP (λ)**	**δ**
HP1	**0.354**		0.875	**0.476**	−0.154	0.836	**0.579**		0.665	**0.613**	*−0.062*	0.647
HP3	**0.725**		0.474	**0.767**	−0.023	0.432	**0.723**		0.478	**0.712**	*0.021*	0.482
HP5	**0.773**		0.403	**0.580**	0.259	0.420	**0.639**		0.591	**0.539**	0.232	0.566
HP6	**0.777**		0.396	**0.832**	−0.025	0.332	**0.720**		0.482	**0.720**	*−0.003*	0.483
HP8	**0.668**		0.554	**0.416**	0.349	0.534	**0.639**		0.591	**0.568**	0.172	0.578
HP10	**0.362**		0.869	**0.532**	−0.209	0.804	**0.564**		0.682	**0.700**	−0.223	0.571
OP2		**0.733**	0.462	−0.034	**0.753**	0.462		**0.598**	0.643	*−0.027*	**0.605**	0.645
OP4		**0.856**	0.267	0.147	**0.758**	0.273		**0.831**	0.310	0.177	**0.753**	0.307
OP7		**0.701**	0.509	0.207	**0.569**	0.495		**0.606**	0.633	0.100	**0.561**	0.635
OP9		**0.708**	0.499	0.157	**0.608**	0.494		**0.577**	0.667	0.175	**0.500**	0.657
OP11		**0.736**	0.458	*0.019*	**0.725**	0.458		**0.733**	0.462	*−0.029*	**0.744**	0.460
OP12		**0.746**	0.444	−0.181	**0.893**	0.360		**0.794**	0.369	−0.210	**0.904**	0.274
Factor correlations and CUs	HP–OP:	0.718		HP–OP:	0.587		HP–OP:	0.427		HP–OP:	0.355	
	OP7–OP9:	0.398		OP7–OP9:	0.389		OP7–OP9:	0.445		OP7–OP9:	0.445	
	HP1–HP10:	0.365		HP1–HP10	0.326		HP1–HP10:	0.434		HP1–HP10:	0.394	
	OP4–OP12	−0.289		OP4–OP12	−0.343		OP4–OP12:	−0.441		OP4–OP12:	−0.460	

*CFA, Confirmatory factor analysis; ESEM, Exploratory structural equation modeling; HP, harmonious passion; OP, obsessive passion; λ, Factor loading; δ, Item uniqueness; CU, correlated uniqueness; Target factor loadings are in bold; Non-significant parameters (p ≥ 0.05) are italicized*.

Both solutions resulted in well-defined factors (ESEM: |λ| = 0.416–0.893, *M* = 0.659; CFA: |λ| = 0.354–0.856, *M* = 0.678). Although cross-loadings were present in the ESEM model (|λ| = 0.154–0.349, *M* = 0.147), these did not undermine the definition of the factors. Moreover, some of the cross-loading are reasonable (e.g., HP10 or OP2), given that they tap into opposing aspects of the target constructs. The three correlated uniquenesses were similar in magnitude for both models and these were also similar to previous studies. The first CU (with the highest modification indices) was between OP7 (i.e., “This activity is the only thing that really turns me on”) and OP9 (i.e., “If I could, I would only do my activity”) which was present in three of the four previous studies mentioned above (Marsh et al., 2013; Chamarro et al., 2015; Zhao et al., 2015). The wording of these items indicated that both refer to the exclusive place that the activity occupies in one's life. The second CU was between HP1 (i.e., “This activity is in harmony with the other activities in my life”) and HP10 (i.e., My activity is in harmony with other things that are part of me) was present for Schellenberg et al. (2014) and Zhao et al. (2015), both belonged to the harmonious passion factor and referred to the fact that the activity was in harmony with other aspects of life. The third CU was between OP4 (i.e., “I have almost an obsessive feeling for this activity”) and OP12 (i.e., “I have the impression that my activity controls me”), and interestingly, despite belonging to the same factor, they had a negative association with each other which might be attributed to the fact that they differentially tap into HP (i.e., OP4 positively, whereas OP12 negatively). As a result of the cross-loadings, factor correlations were also reduced for the ESEM (*r* = 0.587) model relative to the CFA (*r* = 0.718). Finally, both factors were reliably in terms of Cronbach's alpha (α_HP_ = 0.801; α_OP_ = 0.883). Although this index is useful when comparing results to previous findings, it tends to be less reliable (Sijtsma, 2009; Rodriguez et al., 2016). Therefore, McDonald's model-based composite reliability coefficient (McDonald, 1970) was also calculated as follows: ω = (Σ|λ_i_|)2/([Σ|λ_i_|]2 + Σδ_ii_) where λ_i_ are the factor loadings and δ_ii_ the error variances and thus it has the advantage, compared to alpha, of taking into account the strength of association between the items and the latent factors (λ_i_) with the specific measurement errors (δ_ii_). Omega also showed adequate model-based reliabilities (ω_HP_ = 0.778; ω_OP_ = 0.867).

### Sample 2: replication the measurement structure of the passion scale

Goodness-of-fit indices associated with Study 2 are reported in the bottom section of Table 1, while parameter estimates can be seen on the right side of Table 2. Again, the models without correlated uniquenesses resulted in bad fit. However, the inclusion of these same correlated uniquenesses as in Study 1 resulted in marginal fit for the CFA solution with only CFI and RMSEA indicating acceptable fit, while all indices were excellent for the ESEM solution. The examination of parameter estimates revealed well-defined factors with the same magnitude (ESEM: |λ| = 0.500–0.904, *M* = 0.660; CFA: |λ| = 0.564–0.831, *M* = 0.667). Cross-loadings were once again small in magnitude (|λ| = 0.003–0.232, *M* = 0.119) and the association between HP and OP was reduced in ESEM (*r* = 0.355) compared to CFA (*r* = 0.427). The reliability of the factors was also highly supported (α_HP_ = 0.821, α_OP_ = 0.846; ω_HP_ = 0.816, ω_OP_ = 0.841). In sum, based on the findings, the ESEM model was retained as it had better model fit, well-defined and reliable factors and reduced factor correlations.

### Measurement invariance

Upon demonstrating the superiority of the ESEM model for this particular scale, we continued by assessing the extent to which this model could be replicated across the two samples and studies (see Table 3) before investigating the effects of gender and age. Although the extended invariance taxonomy is exhaustive, we only interpret the key models (see Appendix 3 for input for these key models). The configural model (Model S1 in Table 3) achieved a satisfactory level of fit to the data, and supported the weak measurement invariance (Model S2 in Table 3) of the model across samples (ΔCFI/TLI ≤ 0.010; ΔRMSEA ≤ 0.015). For strong invariance (Model S5 in Table 3), although changes in CFI were marginal in relation to cut-off values (ΔCFI = −0.011), changes in TLI (ΔTLI = −0.005), and RMSEA (ΔRMSEA = +0.003) were acceptable. Nevertheless, we explored a model of partial strong invariance involving the relaxation of equality constraints for a single item's (HP5) intercept through the examination of modification indices of the strong invariance model. This model of partial strong invariance (Model S5p) was supported by the data, as well as the remaining models of strict (Model S7) and latent-variance-covariance (Model S9) invariance (ΔCFI/TLI ≤ 0.010; ΔRMSEA ≤ 0.015). Overall, these results confirm that the model was well-replicated across samples. The invariance of latent means was again marginal in relation to typical guidelines (ΔCFI = −0.011; TLI = −0.010; ΔRMSEA = +0.003), thus we opted to probe these differences. In these cases, the latent means of the referent group are constrained to zero (for the purposes of identification), while freely estimated in the other groups, thus providing a direct estimation of group-based differences, estimated in SD units. When the means of Sample 1 were constrained to zero, the means of Sample 2 proved to be higher on both HP (+1.077, *p* < 0.001) and OP (+0.857, *p* < 0.001). The observed differences could be attributed to the fact that, in Study 2, participants had to indicate an activity that they were passionate about, whereas in Study 1, the activities were provided beforehand.

**Table 3:** Tests of measurement invariance for the final retained model across the two studies.

**Model**	**Invariant parameters^a^**	**χ^2^**	**df**	**CFI**	**TLI**	**RMSEA**	**90% CI**	**Comparison model**
Model S1	–	1783.921	83	0.952	0.924	0.072	0.069–0.075	—
Model S2	1	1927.579	103	0.949	0.935	0.067	0.064–0.069	S1
Model S3	1,3	2305.691	115	0.939	0.930	0.069	0.067–0.072	S1, S2
Model S4	1,4	1988.518	106	0.947	0.934	0.067	0.064–0.069	S1, S2
Model S5	1,2	2317.501	113	0.938	0.928	0.070	0.068–0.082	S1, S2
Model S5p	1,2	2250.253	112	0.940	0.930	0.069	0.067–0.072	S1, S2
Model S6	1,3,4	2403.082	118	0.936	0.929	0.070	0.067–0.072	S1, S2, S3, S4
Model S7	1,2,3	2640.170	124	0.930	0.925	0.071	0.069–0.074	S1, S2, S3, S5
Model S8	1,2,4	2309.357	115	0.939	0.930	0.069	0.067–0.072	S1, S2, S4, S5
Model S9	1,2,3,4	2738.334	127	0.927	0.924	0.072	0.070–0.074	S1–S8
Model S10	1,2,5	2651.469	114	0.929	0.918	0.075	0.072–0.077	S1, S2, S5
Model S11	1,2,3,5	3048.584	126	0.918	0.914	0.076	0.074–0.079	S1, S1, S3, S5, S7, S10
Model S12	1,2,4,5	2719.252	117	0.927	0.918	0.075	0.072–0.077	S1, S2, S4, S5, S6, S10
Model S13	1,2,3,4,5	3148.212	129	0.916	0.914	0.077	0.074–0.079	S1–S12

χ^2^, Robust chi-square test of exact fit; df, Degrees of freedom; CFI, Comparative fit index; TLI, Tucker-Lewis index; RMSEA, Root mean square error of approximation; 90% CI, 90% confidence interval of the RMSEA;

a *Parameters that are invariant on that particular level are indicated with a number and are based on the taxonomy of Marsh et al. (2009); see also Morin et al. (2013); 1, invariant factor loadings; 2, invariant item intercepts; 3, invariant item uniquenesses; 4, invariance factor variances and covariances; 5, invariant latent factor means*.

In the following step, we addressed the issue of gender and age effects on the combined sample. Considering gender (Table 4) and age (Table 5) groups separately, complete invariance (loadings, intercepts, uniquenesses, latent variances-covariances, and latent means) was achieved in both cases as apparent by the small changes in fit indices (ΔCFI/TLI ≤ 0.010; ΔRMSEA ≤ 0.015). These results confirm the equivalence of ratings on the Passion Scale and support its use in gender or age groups (when divided into discrete categories). In the next step, we performed the same analytic sequence with the interaction of gender and age groups (3 × 2 = 6 groups). Again, we only interpret the key elements of the taxonomy (see Table 6). Both the configural (Model GA1 in Table 6) and the weak (Model GA2 in Table 4) invariance models were satisfactory in terms of model fit and relative change in fit. Next, strong invariance (Model GA5 in Table 6) was tested which was not achieved (ΔCFI = −0.012; TLI = −0.005; ΔRMSEA = +0.003), potentially suggesting differential item functioning. Again, partial invariance models were pursued and the equivalence constraint of a single item (HP8) was freed in all groups. This relaxation led to acceptable changes (ΔCFI = −0.008; TLI = −0.002; ΔRMSEA = +0.001) when comparing the strong and weak models. The remaining models of strict (Model GA7), latent-variance-covariance (Model GA9), and latent means (Model GA13) invariance (ΔCFI/TLI ≤ 0.010; ΔRMSEA ≤ 0.015). Overall, these results further confirm invariance of measurements by gender and age groups.

**Table 4:** Tests of measurement invariance for the final retained model across gender groups.

**Model**	**Invariant parameters^a^**	**χ^2^**	**df**	**CFI**	**TLI**	**RMSEA**	**90% CI**	**Comparison model**
Model G1	–	1851.820	83	0.954	0.927	0.073	0.070–0.076	—
Model G2	1	1925.055	103	0.953	0.940	0.067	0.064–0.069	G1
Model G3	1,3	1959.804	115	0.952	0.945	0.063	0.061–0.066	G1, G2
Model G4	1,4	1945.835	106	0.953	0.941	0.066	0.063–0.069	G1, G2
Model G5	1,2	2047.581	113	0.950	0.942	0.066	0.063–0.068	G1, G2
Model G6	1,3,4	1985.973	118	0.952	0.946	0.063	0.061–0.066	G1, G2, G3, G4
Model G7	1,2,3	2080.482	125	0.950	0.947	0.063	0.060–0.065	G1, G2, G3, G5
Model G8	1,2,4	2067.585	116	0.950	0.943	0.065	0.063–0.067	G1, G2, G4, G5
Model G9	1,2,3,4	2106.087	128	0.949	0.947	0.062	0.060–0.065	G1–G8
Model G10	1,2,5	2070.241	115	0.950	0.942	0.065	0.063–0.068	G1, G2, G5
Model G11	1,2,3,5	2102.791	127	0.949	0.947	0.063	0.060–0.065	G1, G1, G3, G5, G7, G10
Model G12	1,2,4,5	2089.606	118	0.949	0.943	0.065	0.062–0.067	G1, G2, G4, G5, G6, G10
Model G13	1,2,3,4,5	2127.695	130	0.948	0.948	0.062	0.060–0.064	G1–G12

χ^2^, Robust chi-square test of exact fit; df, Degrees of freedom; CFI, Comparative fit index; TLI, Tucker-Lewis index; RMSEA, Root mean square error of approximation; 90% CI, 90% confidence interval of the RMSEA;

a *Parameters that are invariant on that particular level are indicated with a number and are based on the taxonomy of Marsh et al. (2009); see also Morin et al. (2013); 1, invariant factor loadings; 2, invariant item intercepts; 3, invariant item uniquenesses; 4, invariance factor variances and covariances; 5, invariant latent factor means*.

**Table 5:** Tests of measurement invariance for the final retained model across age groups.

**Model**	**Invariant parameters^a^**	**χ^2^**	**df**	**CFI**	**TLI**	**RMSEA**	**90% CI**	**Comparison model**
Model A1	–	1846.501	126	0.956	0.930	0.072	0.069–0.075	—
Model A2	1	2022.130	166	0.952	0.943	0.065	0.062–0.067	A1
Model A3	1,3	2112.481	190	0.951	0.948	0.062	0.059–0.064	A1, A2
Model A4	1,4	2043.195	172	0.952	0.945	0.064	0.062–0.066	A1, A2
Model A5	1,2	2407.249	186	0.943	0.939	0.067	0.065–0.069	A1, A2
Model A6	1,3,4	2132.086	196	0.950	0.950	0.061	0.059–0.063	A1, A2, A3, A4
Model A7	1,2,3	2500.966	210	0.941	0.944	0.064	0.062–0.066	A1, A2, A3, A5
Model A8	1,2,4	2427.903	192	0.942	0.941	0.066	0.064–0.069	A1, A2, A4, A5
Model A9	1,2,3,4	2520.412	216	0.941	0.946	0.063	0.061–0.066	A1–A8
Model A10	1,2,5	2452.164	190	0.942	0.939	0.067	0.065–0.069	A1, A2, A5
Model A11	1,2,3,5	2546.055	214	0.940	0.945	0.064	0.062–0.066	A1, A1, A3, A5, A7, A10
Model A12	1,2,4,5	2473.565	196	0.941	0.941	0.066	0.064–0.068	A1, A2, A4, A5, A6, A10
Model A13	1,2,3,4,5	2566.222	220	0.940	0.946	0.063	0.061–0.066	A1–A12

χ^2^, Robust chi-square test of exact fit; df: Degrees of freedom; CFI, Comparative fit index; TLI, Tucker-Lewis index; RMSEA, Root mean square error of approximation; 90% CI, 90% confidence interval of the RMSEA;

a *Parameters that are invariant on that particular level are indicated with a number and are based on the taxonomy of Marsh et al. (2009); see also Morin et al. (2013); 1, invariant factor loadings; 2, invariant item intercepts; 3, invariant item uniquenesses; 4, invariance factor variances and covariances; 5, invariant latent factor means*.

**Table 6:** Tests of measurement invariance for the final retained model across gender × age groups.

**Model**	**Invariant parameters^a^**	**χ^2^**	**df**	**CFI**	**TLI**	**RMSEA**	**90% CI**	**Comparison model**
Model GA1	–	2001.350	255	0.955	0.931	0.072	0.069–0.075	—
Model GA2	1	2287.762	355	0.951	0.945	0.064	0.062–0.067	GA1
Model GA3	1,3	2491.000	415	0.947	0.949	0.061	0.059–0.064	GA1, GA2
Model GA4	1,4	2333.175	370	0.950	0.946	0.063	0.061–0.066	GA1, GA2
Model GA5	1,2	2807.866	405	0.938	0.940	0.067	0.065–0.069	GA1, GA2
Model GA5p	1,2	2634.707	400	0.943	0.943	0.065	0.063–0.067	GA1, GA2
Model GA6	1,3,4	2541.553	430	0.946	0.950	0.061	0.059–0.063	GA1, GA2, GA3, GA4
Model GA7	1,2,3	2842.933	460	0.939	0.947	0.062	0.060–0.065	GA1, GA2, GA3, GA5
Model GA8	1,2,4	2679.594	415	0.942	0.945	0.064	0.062–0.066	GA1, GA2, GA4, GA5
Model GA9	1,2,3,4	2893.297	475	0.938	0.948	0.062	0.060–0.064	GA1–GA8
Model GA10	1,2,5	2699.002	410	0.941	0.943	0.065	0.063–0.067	GA1, GA2, GA5
Model GA11	1,2,3,5	2906.761	470	0.938	0.947	0.063	0.060–0.065	GA1, GA1, GA3, GA5, GA7, GA10
Model GA12	1,2,4,5	2743.220	425	0.941	0.945	0.064	0.062–0.066	GA1, GA2, GA4, GA5, GA6, GA10
Model GA13	1,2,3,4,5	2955.778	485	0.937	0.948	0.062	0.060–0.064	GA1–GA12

χ^2^, Robust chi-square test of exact fit; df, Degrees of freedom; CFI, Comparative fit index; TLI, Tucker-Lewis index; RMSEA, Root mean square error of approximation; 90% CI, 90% confidence interval of the RMSEA;

a *Parameters that are invariant on that particular level are indicated with a number and are based on the taxonomy of Marsh et al. (2009); see also Morin et al. (2013); 1, invariant factor loadings; 2, invariant item intercepts; 3, invariant item uniquenesses; 4, invariance factor variances and covariances; 5, invariant latent factor means*.

### Differential item functioning

Although tests of measurement invariance provide a rigorous method for testing, it is less practical for continuous variables such as age. Therefore, we incorporated the linear and quadratic age effects in the final invariance model (Model GA13) and contrasted three competing models. The null model (MM1 in Table 7) provided good fit to the data, but the saturated model (MM2 in Table 7) showed a substantial improvement in model fit relative to the null model (ΔCFI = +0.014; TLI = −0.003; ΔRMSEA = +0.001), indicating that age has an effect on the responses to the Passion Scale. However, the factors-only model (MM3 in Table 4)—where the relations from the predictors to the factors were freely estimated, but not to the items—resulted in a marginal decreased fit (ΔCFI = −0.013; TLI = +0.001; ΔRMSEA = −0.001), suggesting that the age effects cannot be fully explained by the effects on the latent variable and that at least some item responses are affected by it. Although TLI and RMSEA have greater relevance in model comparisons due to the incorporation of correction for parsimony, we investigated DIF through modification indices which suggested that DIF is most likely associated with HP8 (the same item that was identified in measurement invariance). Allowing the direct effects from the predictors to this item resulted in comparable fit to the saturated model (ΔCFI = −0.008; TLI = +0.005; ΔRMSEA = −0.002).

**Table 7:** MIMIC and hybrid Multigroup-MIMIC models.

**Model**	**Description**	**χ^2^**	**df**	**CFI**	**TLI**	**RMSEA**	**90% CI**	**Comparison model**
**STANDARD MIMIC MODEL**								
MM1.	null	2697.062	178	0.939	0.938	0.060	0.058–0.062	—
MM2.	saturated	2065.325	130	0.953	0.935	0.061	0.059–0.063	MM1
MM3.	factors–only	2633.681	170	0.940	0.936	0.060	0.059–0.062	MM2
MM3p.	partial factors–only	2439.893	166	0.945	0.940	0.059	0.057–0.061	MM2
**HYBRID MULTIGROUP AND MIMIC MODEL**								
HY1.	null	3345.094	629	0.934	0.943	0.057	0.055–0.059	—
HY2.	saturated	2641.023	485	0.948	0.942	0.058	0.056–0.060	HY1
HY3.	factors–only	3257.657	605	0.936	0.943	0.057	0.056–0.059	HY2
HY4p.	partial factors–only	3191.998	593	0.937	0.943	0.057	0.056–0.059	HY2
HY5.	invariant DIF	3230.725	603	0.936	0.943	0.057	0.055–0.059	HY4p
HY6.	invariant factors–only	3274.683	623	0.936	0.944	0.057	0.055–0.059	HY5

*MIMIC, Multiple indicators multiple causes model; χ^2^, Robust chi-square test of exact fit; df, Degrees of freedom; CFI, Comparative fit index; TLI, Tucker-Lewis index; RMSEA, Root mean square error of approximation; 90% CI, 90% confidence interval of the RMSEA; DIF, differential item functioning*.

### The hybrid model of the multigroup and mimic models

So far, we have seen that the two different methods with which DIF could be identified converge to the same result, supporting their cross-validation. However, as with the multigroup analyses in relation to information loss (as discussed above), there are inherent disadvantages of the MIMIC approach as it rests on the assumption of strict measurement invariance. Moreover, it lacks the ability to test the invariance of other parameters of a model (i.e., factor loadings or uniquenesses). Therefore, to address the shortcomings of both methods, on the basis of Marsh et al. (2013), we combine these approaches into a single hybrid model by adding the linear and quadratic MIMIC age effects (MM3p of Table 7) to the final six-group model (Model GA13 of Table 6).

As the first step, we estimated a null model (see HY1 in Table 7) which will serve as a baseline comparison. This null model, similar to the standard MIMIC one, posited that there are no MIMIC age effects. This model had adequate fit. The second, saturated model (HY2 in Table 7) had paths from the predictors to the items freely estimated. The comparison of these models reveals whether information was lost in forming discrete age categories instead of using it as a continuous variable. The differences between the two models were negligible with the parsimony-corrected indices remaining stable over the two models (ΔTLI = −0.001; ΔRMSEA = +0.001), suggesting that the MIMIC model does not contribute much beyond the multigroup model with discrete categories.

**Figure 2:**
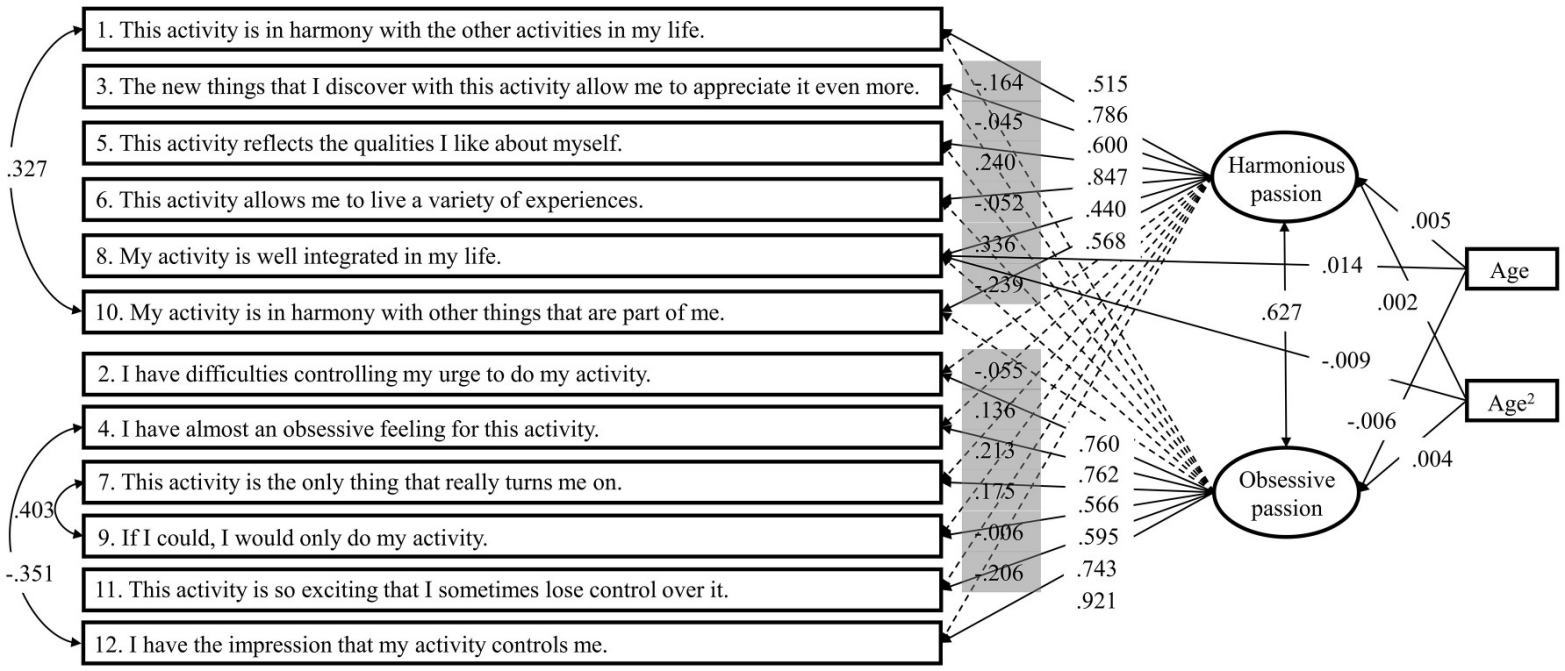
The final hybrid model. In the case of factor loadings, loadings with full arrows, and white background indicate target loadings, whereas number with dashed arrows, and gray background indicate cross-loadings. One-headed arrows represent regression paths, two-headed arrows represent correlations. All parameters are standardized and invariant across the six groups.

Next, we only included the direct age and age^2^ effects on the latent means (HY3 in Table 7). We then added the direct path from the predictors to the item identified in the MIMIC model (HY4p in Table 7) and evaluated whether these were invariant across the six groups (HY5 in Table 7). The changes in fit indices again remained stable, indicating the equivalence of these paths across the different combinations of gender × age. In the final model, we constrained the age and age^2^ effects to be equal in all groups. Once again, relative changes in fit indices were stable, suggesting that the generalizability of the relations between age, age^2^, HP, and OP across gender and age groups. These results revealed that, while age does not have an effect on HP (age: β = 0.005, *p* = 0.067; age^2^: β = 0.002, *p* = 0.213), OP shows a slight and linear decrease (age: β = −0.006, *p* < 0.050) with a small non-linear component (age: β = 0.004, *p* < 0.050) also being significant. Finally, HP8 showed a slight linear increase (β = 0.014, *p* < 0.001) with a negative non-linear component (β = −0.009, *p* < 0.001). The final hybrid model is presented in Figure 2.

## Discussion

The purpose of the present investigation was to illustrate the applicability of the novel ESEM framework on the Passion Scale—the only instrument designed specifically to measure passion—with two independent samples. Our research fits well with the increasing amount of research on ESEM (for an overview, see Marsh et al., 2014) in that the comparison of alternative solutions revealed that ESEM substantially fit the data better than its traditional CFA counterpart and subsequently resulted in a more realistic representation. We also successfully extended the basic ESEM model with tests of measurement invariance, differential item functioning, and a hybrid model incorporating the two approaches to illustrate its flexibility of this sound framework. We now address in turn each of our results and their implications.

As argued in the introduction and demonstrated in this research, CFA might often be considered insufficient as a result of the overly restrictive assumption that items should only load on their corresponding factors, but not on other, conceptually-relevant ones. On the basis of previous studies in the field of SDT (e.g., Howard et al., 2017; Litalien et al., 2017) and specially in relation to passion (e.g., Marsh et al., 2013; Schellenberg et al., 2014), ESEM was expected to overcome the limitations related to the overly restrictive CFA both in terms of unsatisfactory goodness-of-fit and inflated factor correlations (e.g., Maïano et al., 2013; Perera, 2015). Our findings on both samples corroborated these expectations. Furthermore, several non-zero cross-loadings were observed that, when remain unexpressed, could undermine the measurement model (as it did so in the CFA solution). However, none of these cross-loadings were large enough to undermine the definitions of the factors. There were items that loaded positively on their respective factors, while negatively on the opposing one (e.g., HP1, HP6, OP2, or OP12) which could be attributed to the fact that although all measure passion for a certain activity, they tap into specific aspects that are unique to either HP or OP. This is justifiable both from the perspective of theory and the wording of the items; moreover, similar phenomena have been described in research on self-concept (Arens and Morin, 2016) or academic motivations (Guay et al., 2015; Tóth-Király et al., 2017c). It is also important and, at the same time, interesting to note that HP5 and HP8 had positive cross-loadings on OP. One possible explanation could be that these items are not capturing the unique aspects of either HP or OP, but rather these are more general, reflecting on the identity component of passion itself. For instance, if one has an OP for gaming, this activity could still be “well-integrated in his/her life (HP8).” Finally, it has to be noted that three CUs were included in the final measurement models that largely correspond with previous studies (Marsh et al., 2013; Schellenberg et al., 2014; Zhao et al., 2015). The first two pairs are likely a result of parallel wording between the items (i.e., exclusive place of the activity and in harmony of other aspects). As for the third one which appears to be specific to this study. One possibility might be that, in Hungary, having an obsessive feeling for an activity is not necessarily considered bad or negative. Still, the examination of cultural effects was outside the scope of this investigation. Nevertheless, the necessity of three CUs for a two-factor scale with 12 items suggest that there might be some issues with the instrument which might warrant a thorough item-level re-examination. These results indicate that passion researchers may consider the possibility of slightly adjusting the wording of these four items of the OP scale and the other two of the HP scale with different synonyms of the relevant words for these to better fit the underlying theoretical background. Ideally, design thinking or A/B testing (Ries, 2011) of alternative synonyms could be tested on smaller samples as this method has already been fruitfully applied in the construction and improvement of social psychological interventions (Yeager et al., 2016). This step could positively contribute to the more precise measurement of the DMP.

At a more practical level, the present investigation also demonstrated the applicability of ESEM when one wants to explore latent mean differences, with the first option being tests of measurement invariance. A particular strength of this approach, as demonstrated, is the possibility to test a wide range of invariance tests, especially if based on an extended taxonomy (Marsh et al., 2009). Here, we highlighted this strength by investigating full measurement invariance (i.e., factor loadings, intercepts, uniquenesses, latent variances–covariances, and latent means) across different subsamples based on gender, age, and their combination and found strong support for the equivalence of the Passion Scale in these groups with no substantial latent mean differences. These findings are in line with Marsh et al. (2013) who also had high levels of invariance across gender and language groups as well as that of Chamarro et al. (2015). However, one limitation of this statistical approach is that items need to be transformed into a smaller number of categories which is particularly problematic for continuous variables such as age (MacCallum et al., 2002).

One potential solution for this issue, and a second option to investigate latent mean differences, is to use a MIMIC model in which continuous variables could be incorporated. It is also more parsimonious relative to the multigroup analyses and can be performed with a sample of moderate size. Yet, only intercept and latent mean invariance can be tested, without addressing the invariance of the other model parameters. In order to counterbalance the shortcoming of both approaches, a hybrid solution (Marsh et al., 2006, 2013) was also explored that combined the MIMIC effects in the multigroup model for a more precise investigation. In the first step, we contrasted the separate multigroup and MIMIC model and these yielded the same results with HP8 appeared to be non-invariant in both cases. However, it has to be noted that the non-invariance of HP8 was only weakly supported. For a more thorough investigation, one should identify non-invariant items via constraining the factor loading and intercept of the first item of each scale and subsequently comparing other items to this referent (Cheung and Rensvold, 1999). The subsequent hybrid combination revealed that the MIMIC part did not contribute much beyond the categorization. Of additional interest, this model also revealed similar results to that of Marsh et al. (2013) and Chamarro et al. (2015); although to a smaller extent, but age had a negative overall effect on OP with a positive non-linear component. Our results also generalized across the six groups (gender × age). It might potentially be attributed to the midlife crisis that people could experience around the ages of 40 and 50; in this case, they might realize that they should spend more time with the activities that they are passionate about, which in turn might lead to small increase as one gets older. However, future studies are needed to uncover these potential effects.

All in all, ESEM proved to be an adequate statistical framework for the Passion Scale via the incorporation of EFA and CFA features. The explicit expression of cross-loadings provides a more accurate estimation of the construct in question and as long as these remain relatively small in magnitude, they do not undermine the definition of the factors. One could argue that as ESEM is less restrictive, it always results in improved model fit. However, even if cross-loadings are seldom present in our measurement model, ESEM still results in unbiased parameter estimates in terms of factor correlations (Asparouhov et al., 2015). Our findings also reinforce the notion that scale items are not perfect indicators of their respective target factors, thus CFA and ESEM models should systematically be contrasted to take into account a systematic type of measurement error related to the fallible nature of indicators, which in turn helps in identifying a better representation of the data. In relation to the Passion Scale, there is a certain degree of overlap between the items, suggesting that some of them might not only tap into one aspect of passion, but both, and that HP and OP might not easily be distinguished in and of themselves, but by the other variables they are associated with. It is also possible that changes could occur between HP and OP as a result of external events (e.g., one might have HP for work, but due to a relationship conflict, [s]he starts to demonstrate signs of OP for work) which might influence the level of HP and OP. Future studies are needed to better understand the nature and the dynamics of HP and OP.

While the ESEM approach is certainly promising, the Bayesian Structural Equation Modeling (BSEM; Muthén and Asparouhov, 2012)—which is similar to ESEM in terms of freely estimating cross-loadings and giving them a small value—recently came under criticism (Stromeyer et al., 2015). The first concern of the authors was that introducing cross-loadings should be interpreted as modeling noise that masks poorly constructed items and thus justifies the use of an improper instrument. In the present case, some of these cross-loadings were reasonable and meaningful in direction (i.e., HP items loaded positively on HP, but negatively on OP and vice versa), while others were not, suggesting that some items might need to be revised to more strongly be associated with their target factors. The second, similar concern referred to the fact that cross-loadings should not be theoretically permissible and researchers should create items and instruments that can adequately capture the target construct without being associated with other, non-target ones. While we agree with the authors in that items should be as precisely constructed as possible and researchers should strive to achieve this precision, completely pure items are rarely present in social sciences. However, if cross-loadings are to be completely disregarded, then EFA—which serves as a basis for ESEM—should also be neglected. Moreover, as Asparouhov et al. (2015) pointed out, even carefully constructed indicators are likely to present at least some degree of true score associations with non-target constructs. The third and final concern pertained to the fact that cross-loadings that are minimal (i.e., close to zero) should not be included in a measurement model as these only artificially reduce the correlations between the factors. Instead, when multicollinearity is present, a bifactor solution (Reise, 2012) should be pursued which might be able to explain the high associations between the factors. This issue could easily be tested with the recently introduced bifactor-ESEM framework (Morin et al., 2016a,b) and already been successfully used (e.g., Fadda et al., 2017; Litalien et al., 2017; Tóth-Király et al., 2017b) in investigating the two sources of construct-relevant psychometric multidimensionality referring to the presence of conceptually-related and global/specific constructs^2^. Overall, while we believe that ESEM should not be used to hide or “partially mend” poor indicators, this framework could still provide a more realistic representation of the constructs at hand.

Although we were able to illustrate the applicability and richness of the ESEM framework with two independent samples, there are some limitations that need to be addressed. Our data was based on cross-sectional and self-reported questionnaires that could be influenced by bias. The findings about small changes in OP could be complemented by longitudinal settings to examine the temporal changes in HP and OP and to investigate the potential personality- and social variables that could influence passion among adults and younger respondents as well. While we conducted a DIF test, we have to note that scale indeterminacy (Wang, 2004; Cheong and Kamata, 2013) might have caused an issue in the interpretation of the findings. Future studies should aim to circumvent these issues with more advanced and sophisticated methods. For instance, the recently developed moderated non-linear factor analysis (MNLFA; Bauer, 2017) combines the strengths and advantages of the multigroup and MIMIC approaches and could be used in future statistical research. Regarding the Passion Scale itself, while it is a short, two-factor instrument, the inclusion of three CUs suggests the scale and the items might need to be thoroughly investigated and potentially improved upon. Regarding the ESEM framework, a relatively large number of parameters need to be estimated, thus smaller sample sizes could lead to decreased precision in model estimation. The current operationalization of ESEM also prevents its direct inclusion in more complex, predictive, or hierarchical models. However, using the ESEM-within-CFA method (Morin et al., 2013), one could easily transform the ESEM solution into the standard CFA framework and could perform the analyses mentioned above. For the present illustration of the ESEM framework, we only used the Passion Scale; other scales may function differently depending on their various properties such as length, the number of items, the number of items per factor or the correlations between the factors (for more examples, see Marsh et al., 2014). As the cut-off values for the fit indices originate from studies with CFA and the basic maximum likelihood estimation, future simulation studies are needed to investigate the functioning of these cut-off values with ESEM and different estimators.

Notwithstanding these limitations, this investigation showed that ESEM as a synergy of EFA and CFA is effective in the psychometric examination of multidimensional instruments and it can also be complemented with or transformed into other modeling approaches. Generally, when one suspects the presence of multidimensionality stemming from the assessment of conceptually-related construct (Morin et al., 2016), then it is possible that the restrictive assumption of CFA is violated, and a comparison of CFA and ESEM models should be performed. Moreover, the latter is preferred if it has better goodness-of-fit, well-defined factors by their target loadings, and meaningfully reduced factor correlations. However, if the psychometric properties are the same in the CFA solution, then that model is preferable as it is more parsimonious. Nevertheless, we believe that ESEM could be a viable and flexible alternative to CFA and, as we demonstrated, could further be fruitfully extended to address substantially important issues.

## Author contributions

IT-K substantially contributed to study design, data gathering, data analyses, interpretation of the results, and manuscript writing; BB, AR, and GO substantially contributed to the data gathering, interpretation of the results, and revising the manuscript. All authors commented on the draft and contributed to the final version, approved the final version of the manuscript, and agreed to be accountable for all aspects of the work.

### Conflict of interest statement

The authors declare that the research was conducted in the absence of any commercial or financial relationships that could be construed as a potential conflict of interest.

## Footnotes

^1^We also tested the two factors separately, as unidimensional constructs to investigate the potential sources of misfit for HP and OP. As for HP, the model with six items showed bad fit to the data (χ^2^ = 1096.933, df = 9, CFI = 0.898, TLI = 0.831, RMSEA = 0.127 [90% CI 0.121–0.134]). The examination of modification indices suggested that the error terms of HP1 and HP10 should be allowed to correlate. This modification corresponded with our main analyses and resulted in substantially improved fit (χ^2^ = 389.793, df = 8, CFI = 0.964, TLI = 0.933, RMSEA = 0.080 [90% CI 0.073–0.087]). We performed the same analyses for OP which yielded unsatisfactory fit (χ^2^ = 1237.426, df = 9, CFI = 0.912, TLI = 0.853, RMSEA = 0.135 [90% CI 0.129–0.142]). Similar to the main analyses, the correlation between the error terms of OP7 and OP9 was freed, resulting in improved fit (χ^2^ = 521.867, df = 8, CFI = 0.963, TLI = 0.931, RMSEA = 0.093 [90% CI 0.086–0.100]). As the overall fit was still borderline in relation to the RMSEA, we freed one more error covariance between OP4 and OP12 based on modification indices. Due to the latter change, model fit was acceptable (χ^2^ = 301.362, df = 7, CFI = 0.979, TLI = 0.955, RMSEA = 0.075 [90% CI 0.068–0.082]).

^2^Separate (unreported) analyses were performed to investigate the presence of the global/specific source of multidimensionality of the Passion Scale. However, the results did not support the need to incorporate a global factor as was apparent by the worse fit indices and the less-defined factors by their respective factor loadings.
