# Supplementary material for: An Illustration of the Exploratory Structural Equation Modeling (ESEM) Framework on the Passion Scale
Source: Front Psychol. 2017 Nov 7;8:1968. doi: 10.3389/fpsyg.2017.01968 (PMC5681952; doi:10.3389/fpsyg.2017.01968)
Supplement: Supplementary file 1 [file Appendix.docx]

***Online Supplements for:***

**An Illustration of the Exploratory Structural Equation Modeling (ESEM) Framework on the Passion Scale**

István Tóth-Király^1,2,*^, Beáta Bőthe^1,2^, Adrien Rigó^2^, Gábor Orosz^2,3^

^1^ Doctoral School of Psychology, ELTE Eötvös Loránd University, Budapest, Hungary

^2^ Institute of Psychology, ELTE Eötvös Loránd University, Budapest, Hungary

^3^ Institute of Cognitive Neuroscience and Psychology, Research Centre for Natural Sciences, Hungarian Academy of Sciences, Budapest, Hungary

*Corresponding author

István Tóth-Király

Institute of Psychology, ELTE Eötvös Loránd University

Izabella utca 46.

Budapest, H-1064, Hungary

E-mail.: [tothkiralyistvan@gmail.com](mailto:tothkiralyistvan@gmail.com); [toth-kiraly.istvan@ppk.elte.hu](mailto:toth-kiraly.istvan@ppk.elte.hu)

Tel.: 00 36 20 582 9949

**Table of Contents:**

Appendix 1. Characteristics of the Samples Used in the Present Research

Appendix 2: Hungarian and original English versions of the Passion Scale

Appendix 3.1: Input for CFA

Appendix 3.2: Input for ESEM

Appendix 3.3. Input for Measurement Invariance Across Gender – Configural

Appendix 3.4. Input for Measurement Invariance Across Gender – Weak

Appendix 3.5. Input for Measurement Invariance Across Gender – Strong

Appendix 3.6. Input for Measurement Invariance Across Gender – Strict

Appendix 3.7. Input for Measurement Invariance Across Gender – Variance-Covariance

Appendix 3.8. Input for Measurement Invariance Across Gender – Latent Mean

Appendix 3.9. Input for Creating Gender × Age Groups

Appendix 3.10. Input for the standard MIMIC models

**APPENDIX 1 | Characteristics of the Samples Used in the Present Research**

| **Sample** | **Activity** | **Sample size** | **Females (%)** | **Age (SD)** |
| --- | --- | --- | --- | --- |
| Study 1  (N = 7,466) | Facebook use | 740 | 543 (73.4%) | 23.40 (6.19) |
|  | series watching | 2,325 | 1,722 (74.1%) | 28.38 (10.16) |
|  | learning new things | 1,198 | 879 (73.4%) | 24.21 (7.90) |
|  | dancing | 207 | 160 (77.3%) | 27.33 (10.03) |
|  | playing Pokémon Go | 1,131 | 566 (50%) | 24.41 (6.48) |
|  | smartphone gaming | 204 | 131 (64.2%) | 25.34 (7.31) |
|  | online gaming | 220 | 50 (22.7%) | 24.94 (6.10) |
|  | sex | 1,441 | 996 (69.1%) | 26.32 (7.18) |
| Study 2  (N = 504) | various, self-written | 504 | 258 (51.2%) | 39.59 (12.03) |

**APPENDIX 2 | Hungarian and original English versions of the Passion Scale**

|  | **Hungarian Version** | **English Version (Vallerand, 2015)** |
| --- | --- | --- |
| Title | Szenvedély Kérdőív | The Passion Scale |
| Instructions | Miközben erre az időtöltésre gondolsz jelöld be az alábbi skála használatával, hogy mennyire értessz egyet az egyes állításokkal! | While thinking of your favorite activity and using the scale below, please indicate your level of agreement with each item. |
| Rating Scale | 1 – egyáltalán nem értek egyet  2 – nem értek egyet  3 – inkább nem értek egyet  4 – egyet is értek, meg nem is  5 – inkább egyetértek  6 – egyetértek  7 – teljes mértékben egyetértek | 1 – not agree at all  2 – very slightly agree  3 – slightly agree  4 – moderately agree  5 – mostly agree  6 – strongly agree  7 – very strongly agree |
| Item 1 (Harmonious Passion) | Ez az időtöltés összhangban van az életem egyéb elfoglaltságaival. | This activity is in harmony with the other activities in my life. |
| Item 2 (Obsessive Passion) | Nehezen tudom kontrollálni az időtöltésre irányuló késztető, sürgető vágyamat. | I have difficulties controlling my urge to do my activity. |
| Item 3 (Harmonious Passion) | Annak köszönhetően, hogy az időtöltés által új dolgokat fedezek fel, még inkább értékelem azt. | The new things that I discover with this activity allow me to appreciate it even more. |
| Item 4 (Obsessive Passion) | Szinte megszállott vagyok ezzel az időtöltéssel kapcsolatban. | I have almost an obsessive feeling for this activity. |
| Item 5 (Harmonious Passion) | Ez az időtöltés kifejezi azokat a tulajdonságokat, amelyeket önmagamban szeretek. | This activity reflects the qualities I like about myself. |
| Item 6 (Harmonious Passion) | Ez az időtöltés lehetőséget ad arra, hogy sokféle élményt szerezzek. | This activity allows me to live a variety of experiences. |
| Item 7 (Obsessive Passion) | Ez az időtöltés az egyetlen, ami igazán felpörget. | This activity is the only thing that really turns me on. |
| Item 8 (Harmonious Passion) | Ez az időtöltés szerves részévé vált az életemnek. | My activity is well integrated in my life. |
| Item 9 (Obsessive Passion) | Ha tehetném, kizárólag ezzel az időtöltéssel foglalkoznék. | If I could, I would only do my activity. |
| Item 10 (Harmonious Passion) | Ez az időtöltés jól megfér az életem többi részével. | My activity is in harmony with other things that are part of me. |
| Item 11 (Obsessive Passion) | Ez az időtöltés annyira izgalmas, hogy néha elvesztem az irányításomat felette. | This activity is so exciting that I sometimes lose control over it. |
| Item 12 (Obsessive Passion) | Az a benyomásom, hogy az időtöltés irányít engem. | I have the impression that my activity controls me. |
| Item 13 (Passion Criteria) | Sok időt töltök ezzel a tevékenységgel. | I spend a lot of time doing this activity. |
| Item 14 (Passion Criteria) | Szeretem ezt az időtöltést. | I like this activity. |
| Item 15 (Passion Criteria) | Ez az időtöltés fontos nekem. | This activity is important for me. |
| Item 16 (Passion Criteria) | Ez a időtöltés számomra egy szenvedély. | This activity is a passion for me. |
| Item 17 (Passion Criteria) | Ez az időtöltés a része annak, aki vagyok. | This activity is part of who I am. |

**APPENDIX 3.1 | Input for CFA**

VARIABLE:

MISSING ARE ALL (9999);

NAMES ARE

sample activity gender age hp1 op2 hp3 op4 hp5 hp6 op7 hp8 op9 hp10 op11 op12 cp13 cp14 cp15 cp16 cp17 Zage Zage2;

USEVARIABLES ARE

hp1 op2 hp3 op4 hp5 hp6 op7 hp8 op9 hp10 op11 op12;

ANALYSIS:

estimator = mlr; ! robust maximum-likelihood estimator

MODEL:

hp BY hp1 hp3 hp5 hp6 hp8 hp10;

op BY op2 op4 op7 op9 op11 op12;

! correlated uniquenesses

op7 WITH op9;

hp1 WITH hp10;

op4 WITH op12;

**APPENDIX 3.2. | Input for ESEM**

VARIABLE:

MISSING ARE ALL (9999);

NAMES ARE

sample activity gender age hp1 op2 hp3 op4 hp5 hp6 op7 hp8 op9 hp10 op11 op12 cp13 cp14 cp15 cp16 cp17 Zage Zage2;

USEVARIABLES ARE

hp1 op2 hp3 op4 hp5 hp6 op7 hp8 op9 hp10 op11 op12;

ANALYSIS:

estimator = mlr; ! robust maximum-likelihood estimator

rotation = target; ! cross-loadings are “targeted” to be zero with the ‘(~0)’ command.

! ESEM factors are identified with the ‘(*1)’ command.

MODEL:

hp BY hp1 hp3 hp5 hp6 hp8 hp10

op2~0 op4~0 op7~0 op9~0 op11~0 op12~0 (*1);

op BY hp1~0 hp3~0 hp5~0 hp6~0 hp8~0 hp10~0

op2 op4 op7 op9 op11 op12 (*1);

! correlated uniquenesses

op7 WITH op9;

hp1 WITH hp10;

op4 WITH op12;

**APPENDIX 3.3. | Input for Measurement Invariance Across Gender – Configural**

VARIABLE:

MISSING ARE ALL (9999);

NAMES ARE

sample activity gender age hp1 op2 hp3 op4 hp5 hp6 op7 hp8 op9 hp10 op11 op12 cp13 cp14 cp15 cp16 cp17 Zage Zage2;

USEVARIABLES ARE

hp1 op2 hp3 op4 hp5 hp6 op7 hp8 op9 hp10 op11 op12;

GROUPING IS gender (1=male 2=female);

ANALYSIS:

estimator = mlr;

rotation = target;

MODEL:

! factor loadings

hp BY hp1 hp3 hp5 hp6 hp8 hp10

op2~0 op4~0 op7~0 op9~0 op11~0 op12~0 (*1);

op BY hp1~0 hp3~0 hp5~0 hp6~0 hp8~0 hp10~0

op2 op4 op7 op9 op11 op12 (*1);

! item intercepts

[hp1-op12];

! item uniqueness

hp1-op12;

! correlated uniquenesses are fixed to be equal with the arbitrary labels in parentheses

hp1 WITH hp10 (c1);

op7 WITH op9 (c2);

op4 WITH op12 (c3);

! factor means

[hp@0]; [op@0];

MODEL FEMALE:

! factor loadings

hp BY hp1 hp3 hp5 hp6 hp8 hp10

op2~0 op4~0 op7~0 op9~0 op11~0 op12~0 (*1);

op BY hp1~0 hp3~0 hp5~0 hp6~0 hp8~0 hp10~0

op2 op4 op7 op9 op11 op12 (*1);

! item intercepts

[hp1-op12];

! item uniqueness

hp1-op12;

! correlated uniquenesses

! factor means

[hp@0]; [op@0];

**APPENDIX 3.4. | Input for Measurement Invariance Across Gender – Weak**

! only the relevant and changing parts are presented here

MODEL:

! factor loadings

hp BY hp1 hp3 hp5 hp6 hp8 hp10

op2~0 op4~0 op7~0 op9~0 op11~0 op12~0 (*1);

op BY hp1~0 hp3~0 hp5~0 hp6~0 hp8~0 hp10~0

op2 op4 op7 op9 op11 op12 (*1);

! item intercepts

[hp1-op12];

! item uniqueness

hp1-op12;

! correlated uniquenesses are fixed to be equal with the arbitrary labels in parentheses

hp1 WITH hp10 (c1);

op7 WITH op9 (c2);

op4 WITH op12 (c3);

! factor means

[hp@0]; [op@0];

MODEL FEMALE:

! factor loadings are set to be invariant

! item intercepts

[hp1-op12];

! item uniqueness

hp1-op12;

! correlated uniquenesses

! factor means

[hp@0]; [op@0];

**APPENDIX 3.5. | Input for Measurement Invariance Across Gender – Strong**

! only the relevant and changing parts are presented here

MODEL:

! factor loadings

hp BY hp1 hp3 hp5 hp6 hp8 hp10

op2~0 op4~0 op7~0 op9~0 op11~0 op12~0 (*1);

op BY hp1~0 hp3~0 hp5~0 hp6~0 hp8~0 hp10~0

op2 op4 op7 op9 op11 op12 (*1);

! item intercepts

[hp1-op12];

! item uniqueness

hp1-op12;

! correlated uniquenesses are fixed to be equal with the arbitrary labels in parentheses

hp1 WITH hp10 (c1);

op7 WITH op9 (c2);

op4 WITH op12 (c3);

! factor means

[hp@0]; [op@0];

MODEL FEMALE:

! factor loadings are set to be invariant

! item intercepts are set to be invariant

! item uniqueness

hp1-op12;

! correlated uniquenesses

! factor means are now freely estimated

[hp*]; [op*];

**APPENDIX 3.6. | Input for Measurement Invariance Across Gender – Strict**

! only the relevant and changing parts are presented here

MODEL:

! factor loadings

hp BY hp1 hp3 hp5 hp6 hp8 hp10

op2~0 op4~0 op7~0 op9~0 op11~0 op12~0 (*1);

op BY hp1~0 hp3~0 hp5~0 hp6~0 hp8~0 hp10~0

op2 op4 op7 op9 op11 op12 (*1);

! item intercepts

[hp1-op12];

! item uniqueness are now set to be equal with the labels (one per item)

hp1-op12 (u1-u12);

! correlated uniquenesses are fixed to be equal with the arbitrary labels in parentheses

hp1 WITH hp10 (c1);

op7 WITH op9 (c2);

op4 WITH op12 (c3);

! factor means

[hp@0]; [op@0];

MODEL FEMALE:

! factor loadings are set to be invariant

! item intercepts are set to be invariant

! item uniqueness

! correlated uniquenesses

! factor means are now freely estimated

[hp*]; [op*];

**APPENDIX 3.7. | Input for Measurement Invariance Across Gender – Variance-Covariance**

! only the relevant and changing parts are presented here

MODEL:

! factor loadings

hp BY hp1 hp3 hp5 hp6 hp8 hp10

op2~0 op4~0 op7~0 op9~0 op11~0 op12~0 (*1);

op BY hp1~0 hp3~0 hp5~0 hp6~0 hp8~0 hp10~0

op2 op4 op7 op9 op11 op12 (*1);

! item intercepts

[hp1-op12];

! item uniqueness are now set to be equal with the labels (one per item)

hp1-op12 (u1-u12);

! correlated uniquenesses are fixed to be equal with the arbitrary labels in parentheses

hp1 WITH hp10 (c1);

op7 WITH op9 (c2);

op4 WITH op12 (c3);

! factor means

[hp@0]; [op@0];

! factor covariances are invariant due to the arbitrary label

hp WITH op (1);

MODEL FEMALE:

! factor loadings are set to be invariant

! item intercepts are set to be invariant

! item uniqueness

! correlated uniquenesses

! factor means are now freely estimated

[hp*]; [op*];

! factor variances constrained to be one (invariant)

hp@1; op@1;

**APPENDIX 3.8. | Input for Measurement Invariance Across Gender – Latent Mean**

! only the relevant and changing parts are presented here

MODEL:

! factor loadings

hp BY hp1 hp3 hp5 hp6 hp8 hp10

op2~0 op4~0 op7~0 op9~0 op11~0 op12~0 (*1);

op BY hp1~0 hp3~0 hp5~0 hp6~0 hp8~0 hp10~0

op2 op4 op7 op9 op11 op12 (*1);

! item intercepts

[hp1-op12];

! item uniqueness are now set to be equal with the labels (one per item)

hp1-op12 (u1-u12);

! correlated uniquenesses are fixed to be equal with the arbitrary labels in parentheses

hp1 WITH hp10 (c1);

op7 WITH op9 (c2);

op4 WITH op12 (c3);

! factor means

[hp@0]; [op@0];

! factor covariances are invariant due to the arbitrary label

hp WITH op (1);

MODEL FEMALE:

! factor loadings are set to be invariant

! item intercepts are set to be invariant

! item uniqueness

! correlated uniquenesses

! factor means constrained to be zero (invariant)

[hp@0]; [op@0];

! factor variances constrained to be one (invariant)

hp@1; op@1;

**APPENDIX 3.9. | Input for Creating Gender × Age Groups**

VARIABLE:

MISSING ARE ALL (9999);

NAMES ARE

sample activity gender age hp1 op2 hp3 op4 hp5 hp6 op7 hp8 op9 hp10 op11 op12 cp13 cp14 cp15 cp16 cp17 Zage Zage2;

USEVARIABLES ARE

hp1 op2 hp3 op4 hp5 hp6 op7 hp8 op9 hp10 op11 op12;

GROUPING IS group (1=m33 2=m66 3=m99 4=f33 5=f66 6=f99);

! Frequencies were examined beforehand and age was cut at 33% and 66%.

! The continuous age variable is recoded into three discrete categories with the IF function.

! New groups were then defined by combining the newly created age categories with gender.

! EQ means “equal”; LT means “less than”; GE means “greater than or equal to.

! This way, no overlapping groups are created.

DEFINE:

IF (gender EQ 1 AND age LT 22) THEN group = 1;

IF (gender EQ 1 AND age GE 22 AND age LT 26) THEN group = 2;

IF (gender EQ 1 AND age GE 26) THEN group = 3;

IF (gender EQ 2 AND age LT 22) THEN group = 4;

IF (gender EQ 2 AND age GE 22 AND age LT 26) THEN group = 5;

IF (gender EQ 2 AND age GE 26) THEN group = 6

! The rest of the input file for the invariance models are as above, only more groups need to be specified.

**APPENDIX 3.10. | Input for the standard MIMIC model**

! For the sake of simplicity, we demonstrate the different MIMIC models on the base ESEM model.

! However, these could easily be integrated into the invariance model.

USEVARIABLES ARE

hp1 op2 hp3 op4 hp5 hp6 op7 hp8 op9 hp10 op11 op12

Zage Zage2; ! MIMIC variables to be included in the model.

GROUPING IS gender (1=male 2=female);

ANALYSIS:

estimator = mlr;

rotation = target;

MODEL:

hp BY hp1 hp3 hp5 hp6 hp8 hp10

op2~0 op4~0 op7~0 op9~0 op11~0 op12~0 (*1);

op BY hp1~0 hp3~0 hp5~0 hp6~0 hp8~0 hp10~0

op2 op4 op7 op9 op11 op12 (*1);

op7 WITH op9;

hp1 WITH hp10;

op4 WITH op12;

!MIMIC paths of the linear and quadratic effect of age with the ON command

hp1-op12 ON Zage@0;

hp1-op12 ON Zage2@0;

hp-op ON Zage@0;

hp-op ON Zage2@0;

! Null model: All paths are constrained to be zero.

! Saturated model: Paths from predictors to items are estimated (without the ‘@0’), but still zero to the factors (with the ‘@0’).

! Factors-only model: Paths from predictors to factors are estimated (without the ‘@0’), but still zero to the factors (with the ‘@0’).
